# Seven Cases of Lithotomy

**Published:** 1875-07

**Authors:** D. S. Brandon

**Affiliations:** Thomasville, Ga.


ATLANTA

MEDICAL AND SURGICAL JOURNAL.

VOL. XIII.] JULY—1875. [NO. 4.

### Original Communications.

### SEVEN CASES OF LITHOTOMY,

### With Comments on the History and Treatment of Calcarious Affections.

BY D. S. BRANDON, M.D., THOMASVILLE, GA.

In this essay I only propose a synopsis of the cases referred to. The bi-lateral operation with A. H. Stevens' prostatic bi-section was performed on six out of the seven. Two of the number were under the age of fifteen, one thirty-five, two (one cut twice) between fifty and sixty, and one, a negro, sixty-seven years old. One death.

CASE 1.—Mr. B., aged 35, of Thomas county, Georgia, was operated on during the summer of 1853, an account of which was published in the *Southern Medical and Surgical Journal* of that year. The stone being large, it was crushed in the effort to extract it. The fragments saved weighed near two ounces. Recovery good. When operated on he was the father of four children, but had no more by his then wife, who lived several years after the operation, or a subsequent one, which he has been married to more than ten years, although the sexual desire has not been impaired.

CASE 2.—Judge H., of Thomas county, Georgia, age 55, a large, robust man, of fine constitution, as well as general health, has been afflicted with stone for two years. Was operated on October 26th, 1865, assisted by Drs. Roberson and Clower. Operation lasted forty minutes, owing to the depth of the perineum. Stone measured two inches in the long circumference. Recovery satisfactory in about thirty days.

CASE 3.—Mr. D., aged fifty-seven; also of Thomas county, Georgia; has suffered with stone for eight or ten years. Had been sounded two or three times prior to the operation without detecting stone. I saw him in October, 1866, and detected the stone for the first time. He was greatly reduced on account of cystitis, for which he had been treated without improvement. An operation was determined on, but it was thought best to give him the benefit of another month, with a view to build him up for the operation. Tonics and demulcent drinks were used for this purpose, but without benefit. It was now a case of life or death. Without an operation he must die; with it he *might not.* Of these facts he was well satisfied, and did not hesitate, preferring to die rather than suffer as he did, *without the prospect of relief.*

The operation was performed about the last of November, 1866, assisted by Drs. Clower and Groover; his attending physician, Dr. Young, being unwell, was not present. The stone was about two and a half inches in circumference the long way. The patient was left in the care of Dr. Young, and promised well for about six days. On the seventh or eighth day he had a chill, followed by fever; the chilly fever recurring at times for several days. In a short time he lapsed into a typhoid condition, from which he never rallied, and died on the nineteenth day after the operation.

CASE 4.—Judge H., the subject of the second operation, has been in good general health and free from stone symptoms ten months after first operation. At times he had been troubled with some tenderness in the perineal region while in the sitting position. In March, 1867, I visited him with a view to ascertain by sounding if there was another stone. It was readily detected, and an operation agreed upon. The Judge called for the almanac to fix the day for the operation by the "signs," and the 31st was chosen. Assisted by Drs. Palmer and Clower, a second stone, about the size of the first, was removed, but of different formation. Recovery about as before. Within the last two or three years he has had suspicious symptoms, and of late has passed blood, with now and then some calcarious formations; so that the trouble may not yet be ended.

CASE 5.—In May, 1861, I was called to see the son of Mr. K., of Brooks county, Georgia, aged thirteen, supposed to have stone; and if so, to operate. The boy had suffered six or eight years, and was consequently small, though his general condition was good. Assisted by Drs. Hitch and Polhill, I removed a stone resembling in shape a druggist's pestle, weighing about half an ounce. His recovery was rapid, having been discharged in about a week.

Case 6.—Master W. M., an orphan, was of Decatur county, Georgia, aged fourteen years; has suffered with stone since childhood; is greatly reduced; weighs about forty-five pounds; has suffered much with cystitis; cannot retain his urine. Stone was readily detected upon sounding, and an operation agreed upon. He was brought to town on the 23d of March, 1873, and the stone was removed by the lateral operation the next day, with assistance of Drs. Turner, Hopkins, Taylor and Cagle. The stone measured 5 4-5 inches in its long circumference, and weighed near two ounces. On account of the size of the stone the opening in the bladder had to be enlarged twice, in order to get the stone through, and then considerable force was necessary to extract it. The bladder was well syringed, the patient put to bed, and a good dose of morphia given. For seven days all promised well, having passed a little urine the natural way; but on the eighth day after the operation a succession of chilly feelings, with fever, set in. This condition of things lasted near a month, the chilly sensations becoming less distinct after about ten days. At the same time the discharge from the wound—which had been healthy—became watery, and the edges of the wound everted. The surface of the gaping wound had a *red, disintegrated* appearance, resembling powdered brick dust, dry at times. To my mind it was a true hospital gangrene.

Five grains quinine, three times daily, with dover's powder at night, was the medical treatment; and a sol. of nitrate of silver, or carbolic acid, daily injected into the wound, constituted the staple of the surgical treatment for most of this time. About the sixteenth day after the operation the nurse informed me that the feces, with the urine, passed the artificial opening together. The next day I satisfied myself that this was true. Treatment was not changed, however. From this time (about the 10th of August) until the last of the month his condition was precarious indeed. The boy became gloomy and his friends gave him up to die, and he knew this.

About this time some abatement of the fever, together with a little returning appetite, indicated a little improvement, though his friends were slow to admit it, as they had determined by this time to take him home, which they did on the night of the 7th of September, sixteen miles by moonlight. He stood the trip well, and the first report I had, about a week after, was to the effect that he was *decidedly* better; the same treatment being kept up. The change to the country, together with the visits of his old neighbors, had a wonderful effect in this case. No other serious trouble was encountered in the case, and he continued to improve, gaining health and strength all the time. Six months after, he came to see me. He weighed about twenty pounds more than when operated on, and perhaps thirty pounds more than when we parted. There was then and is now a cysto-rectal fistula, which I propose to close up by appropriate treatment, which he has agreed to. He does ordinary farm work all the time now, and is getting quite stout.

CASE 7.—David Bulloch, a negro, of Leon county, Florida, aged sixty-seven; of good health and vigor for a man of his age, although he has had *spasmodic suffering* with stone for nine years. I sounded him in my office the 22d of February of this year—1875. A stone was readily detected. On the 3d of March, aided by Drs. Gardner, Wheeler and Carn, I operated on Davy at his Florida home. The stone, a good sized one, was readily grasped, but being very soft, it crushed on closing the forceps. The larger fragments were removed with the forceps, but the *debris* (one-third, perhaps) was washed out with a Davidson syringe. The horn from off the end of a gum elastic catheter was also removed. It had been there about three months. In a great hurry, at night, Dave introduced the catheter wrong end foremost, and in this way he had lost it in the bladder. My patient, at last accounts, was about well.

The history of urinary calculi presents some peculiarities, that I propose to notice in connection with the cases reported. It is not known that any nation or people are absolutely free from it, but there is a marked difference as to its frequency in various latitudes and among different races, and in the same race in different regions or latitudes. As a general rule the inhabitants of hot and cold countries are alike free from calcarious affections, but there are exceptions to this rule.

India, with much of her territory embraced within the tropics, and Russia, with much of hers within the frigid zone, furnish among their people frequent examples of stone in the bladder.

All other hot and cold climates, so far as the writer knows, are about alike free from troubles of this sort.

In different localities in the same latitude in our own country, the frequency with which we meet with stone is marked and distinctive, while other sections are almost free from such formation. My opinion is that the chemical composition of the *great body* of these formations indicates the *locality* and for the most part the latitude in which they are formed.

In warm countries, with a light soil and *soft water*, most of these formations will be of the *softer* class; and where the opposite condition obtains the *harder* varities will be found most frequent.

Of the seven reported cases above there was but one of the harder class—thought to be lithic acid—the last one in case two, (second operation.)

While none have been subjected to chemical analysis, it is clear that the phosphatic element predominates in six out of the seven. The same analogy holds good with reference to the great body of stone collections in this country, as compared with European collections. The oxalates and the lithic acid stones are more common abroad, and the phosphatic formations are generally met with in this country.

The valleys east of the Mississippi river, covering the Alleghany ridge, with two hundred miles south of that ridge, embracing about as much as five States as large as Georgia, has, it is believed, produced more cases of stone than all the balance of the Union of States. The reports of Dudley, Gross, Eve and others, plainly indicate the truth of the statement. B. F. Dudley cut for stone 225 times; S. D. Gross 115 times; P. F. Eve 106 times; Mettaur, of Virginia, 79 times. There are perhaps a dozen or more beside those mentioned, resident in the territory indicated, that have operated from ten to fifty times, say an average of thirty times each. This will add 360 more to the list, making 885 cases that have been cut at home within the last forty years. Beside this many have sought relief in New York, Philadelphia, Baltimore and Cincinnati, from the area of territory referred to, making a total of not less than 1,500 cases in all, perhaps. From general knowledge, (without the aid of statistics) I feel safe in saying that these estimates are not overdrawn; and I also feel safe in saying that the balance of the Union has not furnished so large a number in the same time.

Assuming that these estimates are true—and they are not far out of the way—the inference is that, in this country at least, the most frequent causes of stone may be looked for in the geological formation of the earth's surface. The entire coast country, from Virginia to the Rio Grande, embracing all the light soil region, where the water, in common parlance, is *soft*, calcarious formations in the human subject are comparatively rare, and those that are met with, as a general rule, are of the softer kinds. While, on the other hand, I think it is true that the Alleghany ridge country, with the contiguous valleys, abound in *hard* water, and hard calculi also.

Another interesting peculiarity in the history of calcarious affections relates to races. The purely negro race enjoys a remarkable immunity from this affection, while the mulatto can claim none of it, if the statistics are correct. As between the white and negro races, the difference, according to Prof. Gross, is about as three to one. Out of one hundred and sixty-one cases that I have collected, the results show eleven negroes and six mulattoes, seventeen in all; a difference of nearly ten to one; or if the mulattoes are not counted, the difference will be about fifteen and a half to one.

The negro race is, and ever will be, an enigma, but not more so in the way of exemptions from disease than in some other particulars. Why they should be less liable to stone affections is as hard to account for as the fact that they will lie and steal almost without exception. The one is, as I believe, a race exemption; the other a race malady, which time or the devices of men will not correct.

Sailors and persons living near the seaside are not often afflicted with stone. For some reason and in some mysterious way the salt atmosphere acts as a prophylactic to such formations. The State of Florida is more perfectly fanned with sea breeze than any other portion of our country, and it is well known that her citizens rarely suffer with stone affections.

The exciting or constitutional causes that in some way produce stone are the rheumatic and gouty habit, which passes through many generations at times. Diseases of the spine, kidneys, bladder, urethra and prostate gland, and a few others of less importance, also give rise to it.

The treatment employed for the removal of stone is mainly surgical; and the cutting operations—lithotomy as now practised is mainly confined to three, the lateral, bi-lateral and median. Of these the first two are the most popular. For myself, I have not been able to see any great advantage that one possesses over either of the other two. The surgeon would do well to stick to the operation he is most familiar with and best prepared to execute.

Within the last forty years Civiale and Dupuytren have popularized the first two of these operations, and they have not lost anything by time, and perhaps will not; but material advances have been made in the construction of instruments for the removal of stone.

In 1824 Civiale published to the world a new plan for the removal of stone, without the use of the knife. To this operation the term lithotrity was applied, and has been and is now practised by some, though not in general favor with the profession. It has been condemned as more difficult to perform, and less safe to the patient, than the cutting operations.

In 1873, Sir Henry Thompson, (than whom no one now living has more reputation in diseases of this kind,) in a paper read before the Midland Medical Society of Birmingham, makes some startling disclosures, in conflict with the history of calcarious affections, both as it relates to age and treatment. At one time this distinguished author gave a tabulated account of 1,827 cases of lithotomy, of which number there were 473 children under five years old; and Civiale, with 5,376 cases, reports 2,314, nearly one-half, below the age of puberty. Statements like these—being hospital reports for the most part—Sir Henry says have missled the profession as to the facts, for the reason that most of such patients are from the poorer classes; but by more careful count, taken from all sources, it will be found "that the well-to-do furnish the majority of stone cases at the other end of life."

Without any statistics on the subject, I am inclined to think there are as many boys living under fifteen as of men over forty years old. If this count be correct, the position taken two years ago by Sir Henry is probably true; and when the statistics come to be properly counted, the disease will be found to be more common among the old than the young. My little experience, as well as observation, attest the truth of the above conclusions.

In the same paper referred to, (a synopsis of which I have seen) Sir Henry Thompson announces the important fact, if fact it be, that stone in the bladder, like many other maladies, is an "exterminable one, so far as it is painful and dangerous;" the inference being, as I understand it, that as all calculi are small at first, if taken at a proper time, may be removed with safety.

Out of sixty-three cases of calculi removed by Sir Henry by the crushing method—the mean ages being more than sixty-five—there was not a *single death;* and he further avers that where the calculi "were *small* he never had yet had a death." If the cutting operations for stone show any results so favorable as this I am not aware of it. So that, if the patient be over the age of puberty, with healthy kidneys, bladder, etc., and particularly if the stone is small, the crushing operation, according to the author referred to, should be preferred. A small sound, that can be readily turned completely round in the bladder, aided by Thompson's or Ferguson's lithotrite, will greatly facilitate the knowledge desired. So that the choice of operations may—and perhaps will at some future day—be determined in all cases by the information obtained as above indicated.

